# Trimetazidine in Chronic Coronary Syndrome and Coronary Artery Disease: A Narrative Review of Clinical Efficacy, Safety, and Current Guideline Position

**DOI:** 10.3390/ph19091370

**Published:** 2026-08-29

**Authors:** Daniel Miron Brie, Cristian Mornoș, Alexandru Tîrziu, Roxana Popescu, Alina Diduța Brie

**Affiliations:** 1 Cardiovascular Disease Institute Timisoara, Gheorghe Adam Street, No. 13A, 300310 Timisoara, Romaniamornos.cristian@umft.ro (C.M.); 2 Research Center of the Institute of Cardiovascular Diseases, Gheorghe Adam Street, No. 13A, 300310 Timisoara, Romania; 3 Department of Cardiology, “Victor Babeș” University of Medicine and Pharmacy Timisoara, Eftimie Murgu Square, No. 2, 300041 Timisoara, Romania; 4 Department of Functional Sciences, “Victor Babeș” University of Medicine and Pharmacy, Tudor Vladimirescu Street, No. 14, 300174 Timisoara, Romania; 5 Center of ImmunoPhysiology and Biotechnologies (CIF-BIOTECH), Department of Functional Sciences, “Victor Babeș” University of Medicine and Pharmacy, Tudor Vladimirescu Street, No. 14, 300174 Timisoara, Romania; 6 Department of Cell and Molecular Biology, “Victor Babeș” University of Medicine and Pharmacy, Tudor Vladimirescu Street, No. 14, 300174 Timisoara, Romania; popescu.roxana@umft.ro (R.P.); alina.brie@umft.ro (A.D.B.); 7 ANAPATMOL Research Center, “Victor Babeș” University of Medicine and Pharmacy, Tudor Vladimirescu Street, No. 14, 300174 Timisoara, Romania; 8 “Louis Țurcanu” Emergency Children Hospital, Doctor Iosif Nemoianu Street, No. 2, 300011 Timisoara, Romania

**Keywords:** trimetazidine, chronic coronary syndrome, stable angina, coronary artery disease, metabolic therapy, antianginal, percutaneous coronary intervention, ischemic cardiomyopathy, ATPCI, ESC guidelines

## Abstract

**Background:** Chronic coronary syndrome (CCS) is a major source of morbidity and healthcare utilization, and a substantial proportion of patients continue to experience angina despite contemporary revascularization and guideline-directed medical therapy. Trimetazidine, a metabolic anti-ischemic agent that does not affect heart rate or blood pressure, has been used for decades as a non-hemodynamic add-on. Its position in international guidelines, however, has shifted. We aimed to synthesize current evidence on the clinical efficacy, safety, and guideline status of trimetazidine in CCS and coronary artery disease (CAD) and to delineate clinical circumstances in which a symptom-directed trial may be considered. **Methods**: We performed a structured narrative review of randomized controlled trials, network and conventional meta-analyses, and large observational studies indexed in PubMed/MEDLINE, Embase, and the Cochrane Library between January 1990 and March 2026. Reference lists of eligible articles, international clinical practice guidelines, and regulatory communications from European and national agencies were also examined. **Results:** Across pooled analyses, trimetazidine modestly but reproducibly improved exercise tolerance and reduced angina frequency and short-acting nitrate use, with efficacy comparable to other non–heart-rate-lowering antianginal agents. Improvements in left ventricular function were reported in small, single-center studies in ischemic cardiomyopathy and selected high-risk subgroups; however, these findings have not been validated in contemporary large-scale trials, and their clinical significance against modern guideline-directed therapy is unknown. The ATPCI trial confirmed safety but showed no reduction in major adverse cardiovascular events after percutaneous coronary intervention. Rare extrapyramidal effects prompted the 2012 European Medicines Agency referral and several prescribing restrictions. **Conclusions:** Trimetazidine remains a reasonable symptom-directed adjunct for residual angina in carefully selected CCS patients, particularly those in whom further hemodynamic up-titration is limited or poorly tolerated. The 2024 ESC class IIb recommendation may partly reflect a broader emphasis on prognostic benefit, although the neutral post-PCI outcome evidence and regulatory safety restrictions also support a more cautious position.

## 1. Introduction

Chronic coronary syndrome (CCS) affects an estimated 100 million adults worldwide and remains one of the largest single causes of morbidity, loss of quality of life, and healthcare utilization in adult cardiology [1,2]. Anatomic revascularization does not solve the problem on its own: roughly one in three patients continues to experience angina one year after percutaneous coronary intervention (PCI), reflecting residual ischemia, microvascular dysfunction, incomplete revascularization, and the metabolic consequences of chronic disease [1,3,4,5,6]. For these patients, the traditional toolkit—β-blockers, calcium channel blockers (CCBs), and long-acting nitrates—is not always sufficient. Hypotension, bradycardia, fatigue, and the limits of dose titration often prevent further hemodynamic optimization, and a non-negligible fraction of patients tolerate poorly any further uptitration.

Trimetazidine offers a mechanistically distinct route to symptom control. By shifting myocardial substrate utilization from fatty acid β-oxidation toward glucose oxidation, it improves ischemic energetics without lowering heart rate, blood pressure, or contractility [7,8,9]. That mechanistic profile has kept the drug in the second-line antianginal armamentarium for three decades, but its place in therapy has been renegotiated several times. Most recently, in the 2024 European Society of Cardiology (ESC) guidelines on chronic coronary syndromes, trimetazidine was downgraded from class IIa—where it had stood under the 2013 and 2019 ESC guidelines [10,11]—to class IIb [1]. The downgrade came after the neutral results of the ATPCI trial [3] and the regulatory restrictions imposed by the European Medicines Agency (EMA) following its 2012 Article 31 referral [12].

Earlier reviews largely drew on pre-PCI or early-PCI-era evidence; here we synthesize data on trimetazidine as an add-on therapy in post-PCI CCS managed under contemporary guideline-directed medical therapy, with the explicit aim of defining what the drug is still useful for in current practice. The review addresses five questions: (i) What is the contemporary efficacy of trimetazidine on symptoms and exercise capacity? (ii) Does it influence left ventricular structure and function? (iii) What does ATPCI tell us about hard outcomes after PCI? (iv) What are the boundaries of safe use in light of the 2012 EMA restrictions and the World Anti-Doping Agency (WADA) listing? (v) In which clinical circumstances a carefully monitored, symptom-directed trial may be reasonable in 2026?

## 2. Methods

This is a structured narrative review; it was not conducted and should not be read as a systematic review. The search and study selection followed a structured, pre-defined strategy, but the protocol was not registered to PROSPERO, no formal risk-of-bias instrument was applied across all included studies, and no quantitative pooling was undertaken (see Section 11). We searched PubMed/MEDLINE, Embase, and the Cochrane Central Register of Controlled Trials from January 1990 to March 2026, combining the terms “trimetazidine”, “chronic coronary syndrome”, “stable angina”, “coronary artery disease”, “ischemic cardiomyopathy”, “heart failure”, and “percutaneous coronary intervention” using Boolean operators (AND, OR). The complete search string used in PubMed is provided in Supplementary Material S1. Reference lists of all included articles and of relevant prior reviews were screened to identify additional eligible studies. International clinical practice guidelines (ESC, AHA/ACC, NICE) and regulatory communications from European and national agencies (EMA, ANSM, MHRA) were also reviewed.

Eligible studies were randomized controlled trials (RCTs), network and conventional meta-analyses, systematic reviews, and large observational cohorts. Inclusion criteria, specified in advance, required, for efficacy studies, enrollment of at least 50 adult patients with CCS, stable CAD, or ischemic heart failure and a follow-up of at least three months; smaller controlled mechanistic or hypothesis-generating studies (≥20 patients) were retained only where they addressed left-ventricular function, viability, or microvascular endpoints not adequately covered by larger trials; and reporting of at least one of the following: exercise tolerance, angina frequency, short-acting nitrate consumption, Seattle Angina Questionnaire (SAQ) or similar patient-reported outcome, left ventricular function or remodeling, major adverse cardiovascular events (MACE), or all-cause mortality. We excluded case reports, case series of fewer than 20 patients, single-arm uncontrolled studies, non-cardiac indications (vertigo, tinnitus, visual disorders), and trials without a clearly defined clinical or functional endpoint. Publications in languages other than English were excluded—a decision that omits a sizeable Chinese-language literature and is acknowledged in the Limitations as one of the most likely sources of bias. It should be noted, however, that several of the pooled meta-analyses on which this review relies (notably the Danchin, Peng, and Lin syntheses) themselves incorporate non-English—particularly Chinese-language—primary trials; the exclusion therefore operates at the level of primary studies rather than the secondary evidence synthesized here.

**Figure 1:**
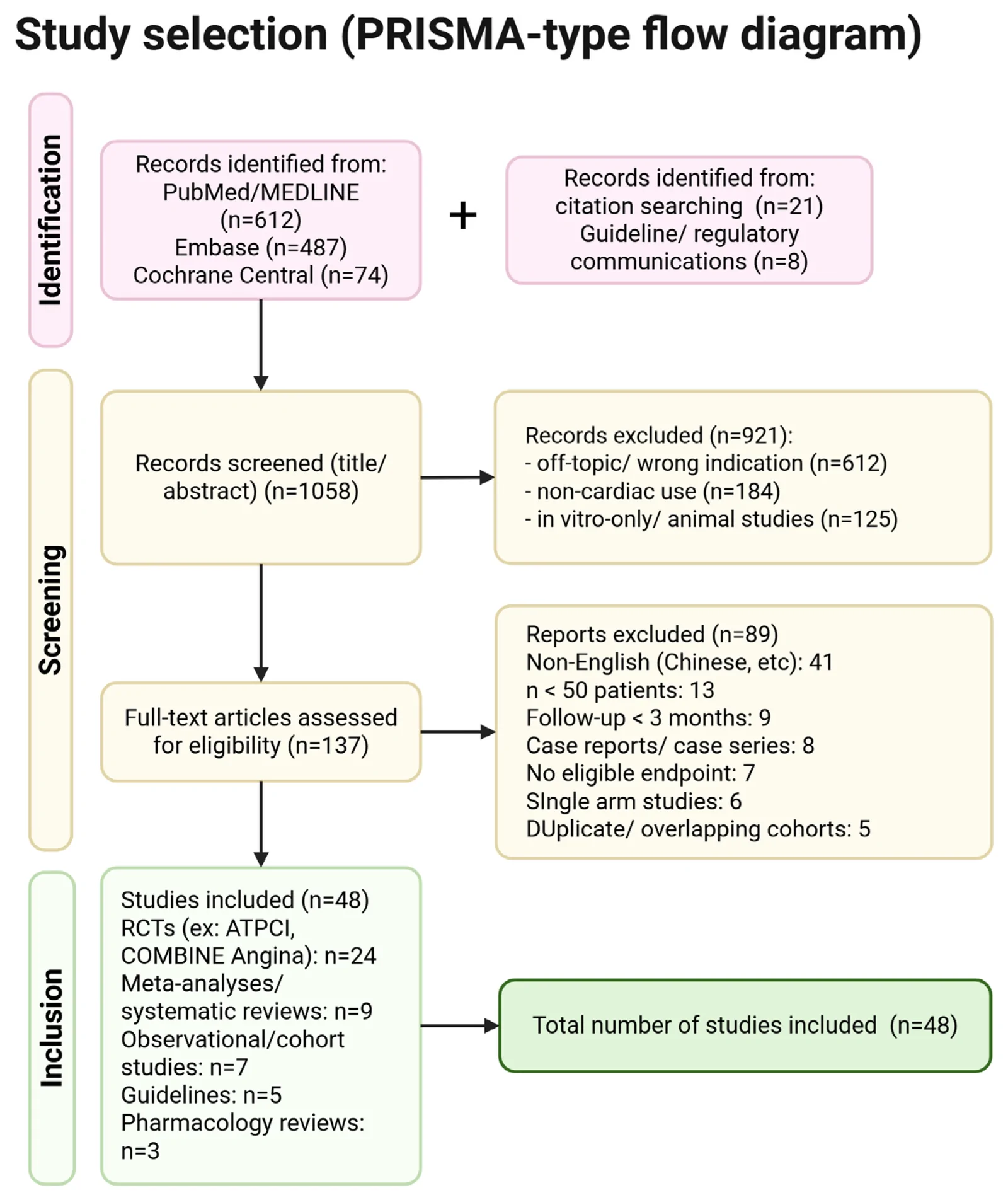
PRISMA-style flow diagram of study identification, screening, and inclusion for this structured narrative review of trimetazidine in chronic coronary syndrome. The diagram is provided to enhance transparency of the literature search and selection process and should not be interpreted as indicating systematic-review methodology. The complete PubMed search string and detailed inclusion/exclusion criteria are provided in Supplementary Material S1.

Where several meta-analyses or overlapping trials covered the same question, we retained the most recent or methodologically stronger work. Titles and abstracts were screened independently by two authors (D.M.B. and A.D.B.); potentially eligible full-text articles were assessed by both reviewers; disagreements were resolved by discussion with a third author (C.M.) when consensus was not reached. A formal risk-of-bias assessment using Cochrane RoB 2.0 was not performed, given the narrative nature of the synthesis and the heterogeneity of designs; instead, methodological quality was appraised qualitatively, with attention to randomization, allocation concealment, blinding, completeness of follow-up, and outcome adjudication, and a summary judgment is provided for each pivotal trial in Table 1. The qualitative assessment on certainty of studies is further described in Table 2. The review protocol was not prospectively registered. The flow of records is shown in Figure 1. The PRISMA-style format is used solely to make the search, screening, and selection process transparent. However, it does not imply that this narrative review used systematic-review methodology or meets PRISMA reporting standards.

## 3. Mechanism of Action and Pharmacological Properties

Trimetazidine (1-[2,3,4-trimethoxybenzyl]piperazine dihydrochloride) is the prototype partial fatty acid oxidation (pFOX) inhibitor. Its molecular target is long-chain 3-ketoacyl-CoA thiolase (3-KAT), the terminal enzyme of mitochondrial fatty acid β-oxidation [7,9]. By partially inhibiting this enzyme, trimetazidine forces the ischemic or failing myocardium to draw a larger share of its ATP from glucose oxidation—a pathway that yields more ATP per molecule of oxygen consumed (approximately 12% more ATP/O_2_) and is therefore preferable when oxygen supply is the limiting factor [7,8].

TMZ increases pyruvate dehydrogenase activity (the enzyme that links glycolysis to the citric acid cycle) and enhances the production of ATP via glucose metabolism [25]. Tan et al. have found in preclinical models of ischemia that trimetazidine treatment reduces the overexpression of TFRC (transferrin receptor) and ACSL4 (acyl-CoA-synthase 4), while favoring FTH (ferritin heavy chain) upregulation. TFRC and ACSL4 favor intracellular iron (Fe^2+^) accumulation, which in turn generates excess hydroxyl radicals responsible for lipid peroxidation via the Fenton reaction. In terms of glutathione metabolism, trimetazidine was found to upregulate GPX4 (glutathione peroxidase) and SLC7A11 while reducing CHAC1 (ChaC glutathione-specific γ-glutamylcyclotransferase 1) levels in myocardial ischemia–reperfusion-treated cells. Furthermore, TMZ improves and attenuates myocardial injury via the sirtuin pathway by restoring Sirt3 and Nrf2 activity. Sirt3 modulates cell death and autophagy, and coordinates mitochondrial redox homeostasis. Its deficiency promotes cardiac fibrosis via p53-mediated acetylation-induced ferroptosis in myofibroblasts [26].

**Figure 2:**
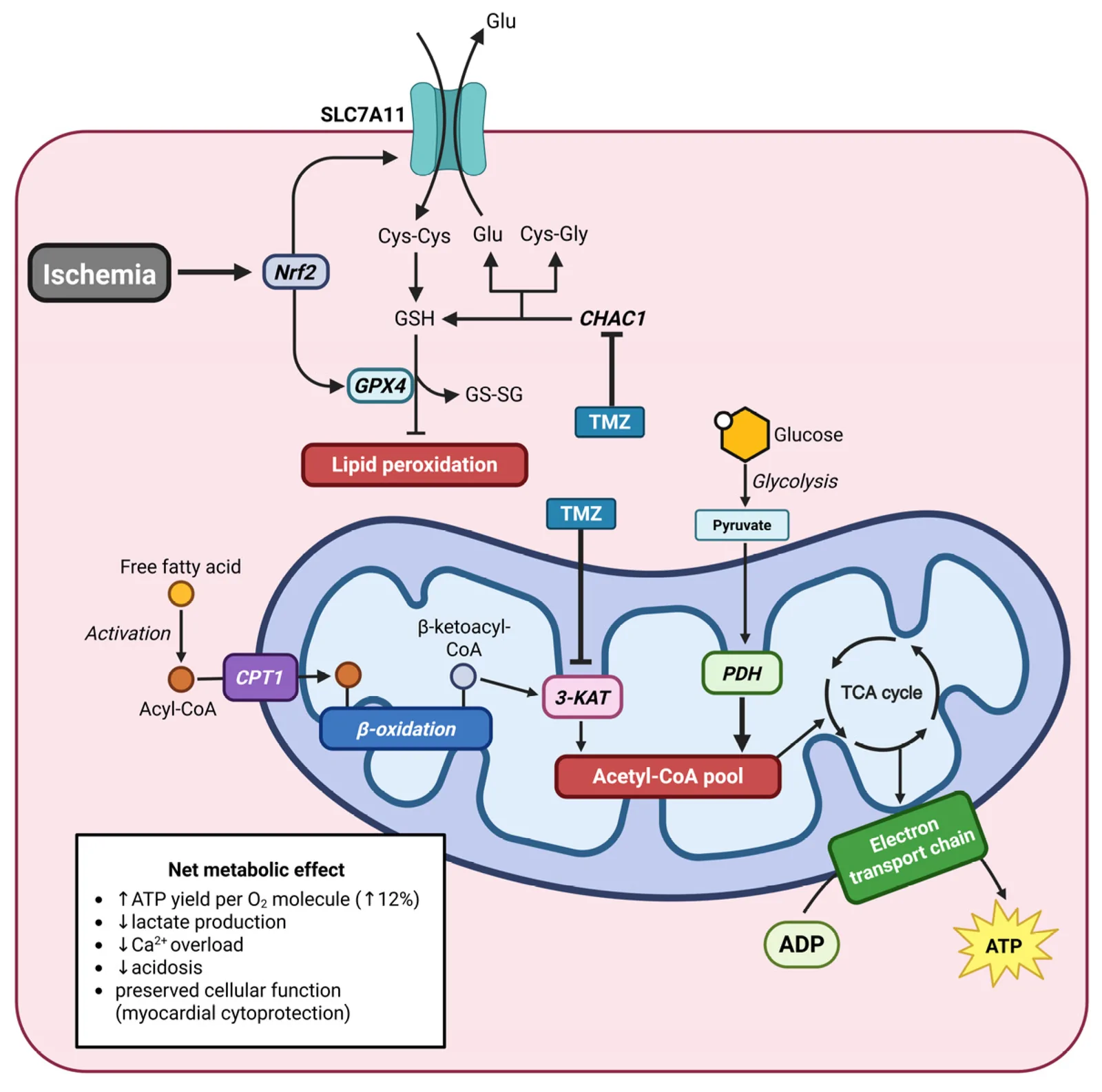
Cardiac substrate metabolism and the site of action of trimetazidine. Selective inhibition of long-chain 3-ketoacyl-CoA thiolase (3-KAT) shifts myocardial substrate use from fatty acid β-oxidation toward glucose oxidation, a more oxygen-efficient pathway under ischemic conditions. CHAC1, ChaC glutathione-specific gamma-glutamylcyclotransferase 1; CPT-1, carnitine palmitoyltransferase 1; GPX4, glutathione peroxidase 4; Nrf2, nuclear factor erythroid 2-related factor 2; PDH, pyruvate dehydrogenase; SLC7A11, Solute Carrier Family 7 Member 11; TCA, tricarboxylic acid cycle; TMZ, trimetazidine; 3-KAT, long-chain 3-ketoacyl-CoA thiolase. Created in BioRender. Tirziu, A. (2026) https://BioRender.com/5l029er (Accessed on 7 August 2026).

TMZ exerts its cardioprotective effects by promoting PINK1/Parkin-mediated mitophagy, rebalancing mitochondrial dynamics, and consequently suppressing GPX4-dependent ferroptosis. TMZ activates the PINK1/Parkin pathway, restoring mitochondrial quality control and promoting the clearance of damaged mitochondria. TMZ treatment upregulates MFN1 and downregulates DRP1, thereby rebalancing mitochondrial dynamics towards a fusion-dominant status. This shift mitigates fission-mediated mitochondrial fragmentation, preserving bioenergetic competence, and suppressing redox imbalance. MFN1 (mitofusin 1) mediates mitochondrial fusion, while DRP1 (dynamin-related protein 1) drives mitochondrial fission [27]. Figure 2 illustrates the mechanism of action of trimetazidine (TMZ).

Under physiological conditions, the heart derives approximately two-thirds of its energy from fatty acid oxidation and the remainder from glucose oxidation. During ischemia, fatty acid oxidation becomes inefficient and produces toxic intermediates—long-chain acyl-CoA, acyl-carnitine, and free fatty acids—which disturb cellular ion homeostasis, uncouple glycolysis from glucose oxidation, and aggravate intracellular acidosis. The metabolic switch induced by trimetazidine improves the coupling between glycolysis and glucose oxidation, limits lactate accumulation, supports intracellular ATP, attenuates calcium overload, and helps preserve cellular function under ischemic stress [7,8,28,29,30].

Beyond this canonical effect, experimental work has documented additional, potentially relevant actions: reduced oxidative stress and lipid peroxidation, preservation of mitochondrial membrane integrity, attenuation of inflammation and neutrophil infiltration in reperfused myocardium, and improved endothelial function [8,14,28,31,32]. Whether any of these contributes meaningfully to clinical effects in humans remains to be further explored.

Crucially, all of this occurs without hemodynamic cost: trimetazidine does not affect heart rate, blood pressure, or contractility in non-ischemic tissue [7,8]. This pharmacological neutrality is precisely what makes the drug useful as an add-on in patients who cannot tolerate further β-blocker or CCB titration. Standard dosing is 35 mg twice daily for the modified-release formulation, 80 mg once daily for the new once-daily modified-release formulation where available, or 20 mg three times daily for the immediate-release form [3,19,33]. Oral bioavailability is good, peak plasma concentrations are reached within 2–5 h, the apparent terminal half-life is approximately 6–7 h, and elimination is predominantly renal as unchanged drug, which is the basis for the dose adjustments shown in Table 3. There are no clinically relevant interactions with antiplatelet agents, statins, β-blockers, calcium channel blockers, or oral anticoagulants [7,28].

## 4. Clinical Efficacy in Stable Angina and Chronic Coronary Syndrome

### 4.1. Exercise Tolerance and Angina Symptoms

The most comprehensive synthesis to date remains the Danchin network meta-analysis, which pooled 218 randomized trials enrolling 19,028 patients with stable angina [13]. Compared with placebo, trimetazidine added a mean of 46 s to total exercise duration (95% credibility interval [CrI]: 28–66 s), 55 s to the time to 1 mm ST-segment depression (CrI: 35–77 s), and 54 s to the time to onset of angina (CrI: 24–84 s). A 46 s gain sits below the 60–90 s figure conventionally cited as the minimal clinically important difference on the Bruce protocol, and that fact should be stated transparently. What lends the signal credibility is its consistency: the same direction of effect emerges across more than 200 trials, accompanies measurable reductions in angina attacks and rescue nitrate use, and translates into improvements on patient-reported instruments such as the SAQ. Effect sizes were largest in trials with a longer treatment duration (≥12 weeks) and in patients with more frequent baseline angina, an observation consistent with mechanistic expectations.

These findings have been replicated by smaller meta-analyses. Marzilli and Klein pooled double-blind RCTs and reported similar effects on exercise capacity and symptoms [14]. Peng et al. analyzed 13 RCTs (1628 patients) and found that trimetazidine added to another antianginal agent reduced angina attacks by 0.95 per week (95% CI: −1.30 to −0.61) and short-acting nitrate use by 0.98 doses per week (95% CI: −1.44 to −0.52), with a gain of 49.8 s in peak exercise duration relative to the comparator [15]. Hu et al., using echocardiographic and radionuclide endpoints, reported parallel improvements in surrogate markers of regional contractility [34]. Individual landmark randomized trials underpin these pooled estimates: the TRIMPOL II and TEMS studies established the antianginal efficacy of trimetazidine added to metoprolol or compared with propranolol [35,36], and its combination with diltiazem produced comparable symptomatic gains without hemodynamic penalty [37]. 

The most recent real-world evidence comes from the COMBINE Angina study [23], a prospective multinational observational program (n = 578 per-protocol) in patients newly diagnosed with stable angina who remained symptomatic on a first-line β-blocker or CCB. Early add-on trimetazidine produced a striking improvement in the Seattle Angina Questionnaire-7 summary score, rising from 39.2 ± 13.7 at baseline to 78.4 ± 16.3 at four months (*p* < 0.0001). Angina frequency, Canadian Cardiovascular Society (CCS) class, and short-acting nitrate consumption all improved, and the rate of treatment-related adverse events was 0.7%. The observational design limits causal inference, and the magnitude of improvement likely reflects in part a regression to the mean and the introduction of any effective add-on therapy in a previously inadequately controlled population; nonetheless, the direction and rapidity of the patient-reported response are consistent with current guideline advice to consider early combination therapy in symptomatic patients [1].

Two caveats temper the symptomatic data. First, most pivotal trials were conducted before the routine use of high-intensity statins [38], sodium–glucose cotransporter 2 (SGLT2) inhibitors [39,40], glucagon-like peptide-1 (GLP-1) receptor agonists [41], contemporary drug-eluting stents, and intensive risk-factor control. First, the incremental effect of trimetazidine in patients on full modern secondary prevention is therefore uncertain and potentially smaller than the historical literature suggests [28]. Second, between-trial heterogeneity in dose, formulation, background therapy, and outcome definitions makes it difficult to translate the pooled estimates into a precise prediction for any individual patient. Publication bias in favor of positive trials in symptomatic agents cannot be excluded and was suggested by funnel plot asymmetry in at least one prior meta-analysis [15].

### 4.2. Comparative Effectiveness with Other Antianginal Agents

When trimetazidine is compared directly with other non–heart-rate-lowering antianginal agents in the Danchin network analysis, the differences disappear: +7 s on exercise duration (95% CI: −12 to 28), −1 s on time to 1 mm ST-segment depression (95% CI: −23 to 22), +8 s on time to onset of angina (95% CI: −22 to 40), and −0.28 angina attacks per week (95% CI: −1.17 to 0.64) [13]. In practical terms, the drug provides symptomatic benefit of roughly the same magnitude as alternative second-line agents while offering a metabolic mechanism that does not add hemodynamic load on top of an already-titrated regimen [7,8]. No head-to-head RCT has formally compared trimetazidine with ranolazine, and the indirect comparisons from network analyses cannot resolve whether one agent should be preferred over the other in routine practice; the antianginal efficacy of ranolazine itself rests chiefly on the CARISA, ERICA, and MERLIN-TIMI 36 programs [42,43,44]—an evidence gap addressed in Section 11.

## 5. Effects on Left Ventricular Function

Evidence of TMZ effect on left ventricular function emerged with a small but provocative 1990 trial by Brottier et al. [16]. Twenty patients with severe ischemic cardiomyopathy (NYHA class III–IV) received trimetazidine 60 mg daily or placebo on top of conventional therapy for six months. Dyspnea improved in all patients on trimetazidine versus one on placebo (*p* < 0.001), residual angina resolved in most, and the need for additional anti-ischemic therapy fell sharply.

Sixteen years later, Fragasso and colleagues delivered a larger and methodologically tighter test [17]. In 55 patients with heart failure (predominantly ischemic), trimetazidine 20 mg three times daily added to conventional therapy increased left ventricular ejection fraction (LVEF) from 36 ± 7% to 43 ± 10% (*p* = 0.002) and reduced left ventricular end-systolic volume from 98 ± 36 mL to 81 ± 27 mL (*p* = 0.04) over a mean follow-up of 13 months. Belardinelli and colleagues, using low-dose dobutamine stress echocardiography, showed that the contractile reserve of chronically dysfunctional myocardium improved during trimetazidine therapy—a marker of preserved viability rather than simply of better hemodynamic loading [18]. These observations have since been extended by metabolic-imaging and randomized data: Tuunanen et al. documented improved cardiac efficiency in idiopathic dilated cardiomyopathy [45], Di Napoli et al. reported sustained functional and anti-inflammatory benefit over 18 months in ischemic dilated cardiomyopathy [46], and Belardinelli et al. showed that trimetazidine potentiates the effects of exercise training on ejection fraction and endothelial function [47].

Meta-analyses by Hu et al., using echocardiography and radionuclide angiography as endpoints, and the broader narrative synthesis by Chierchia et al. reach the same qualitative conclusion: improvements in left ventricular function were reported across these small, pre-modern-therapy studies [34,48]. Larger pooled analyses across randomized trials in chronic heart failure are concordant, reporting gains in left ventricular ejection fraction and reductions in cardiac hospitalization [49,50]. However, no contemporary large-scale randomized trial has tested trimetazidine’s effect on ventricular function against a background of current guideline-directed heart failure therapy (sacubitril/valsartan, SGLT2 inhibitors, high-intensity statins, contemporary revascularization), and the clinical significance of these signals in present-day practice therefore remains unestablished.

None of this rises to the level of a disease-modifying claim. The studies are small, heterogeneous, single-center, and rely on surrogate echocardiographic endpoints rather than on cardiovascular death or heart-failure hospitalization. They are also drawn predominantly from an era before the routine use of sacubitril/valsartan, mineralocorticoid receptor antagonists in HFrEF, and SGLT2 inhibitors across the ejection-fraction spectrum [39,51]. The signals are best read as hypothesis-generating and as a motivation for adequately powered modern trials in defined ischemic-cardiomyopathy phenotypes—not as evidence for routine use in heart failure outside the context of residual ischemia.

## 6. Trimetazidine in Special Populations

### 6.1. Diabetic Patients with Coronary Artery Disease

Diabetes mellitus shifts cardiac substrate use toward fatty acid oxidation and away from glucose oxidation, exactly the metabolic pattern that trimetazidine is designed to reverse [8,28]. Lin et al. conducted a meta-analysis of RCTs in patients with diabetic CAD and reported beneficial effects on a composite of cardiovascular outcomes [24]. In a double-blind, placebo-controlled study, Rosano et al. similarly reported improved systolic and diastolic function in diabetic patients with ischemic cardiomyopathy [52]. The most informative single trial is by Xu et al., who randomized 510 elderly patients with multivessel CAD and type 2 diabetes after drug-eluting stent implantation to trimetazidine 20 mg three times daily or control [19]. Over two years of follow-up, the trimetazidine arm experienced fewer episodes of angina (*p* = 0.024) and silent myocardial ischemia (*p* = 0.009) and a better angina-free survival (*p* = 0.011). Left ventricular structure and function remained essentially stable in the trimetazidine group, whereas they deteriorated in the control group (all *p* < 0.01). The trial is a single-center, open-label study, and enrolled a population at very high metabolic risk; the direction and consistency of the effect nonetheless mirror what mechanistic considerations would predict. These findings are hypothesis-generating and require prospective confirmation in adequately powered multicenter studies. However, they do not establish preferential clinical benefit in diabetic CAD.

### 6.2. Elderly Patients

Older patients are more likely to tolerate hemodynamic antianginals poorly. Orthostatic hypotension, baseline bradycardia, conduction-system disease, frailty, and a higher burden of polypharmacy all narrow the therapeutic window of β-blockers and CCBs. The TIGER study addressed this group directly and reported favorable efficacy and tolerability of modified-release trimetazidine 35 mg in geriatric stable angina [20]. Lin et al. described similar findings in a separate elderly CAD cohort [53]. The absence of a hemodynamic effect may make trimetazidine an option worth considering in selected older patients; however, age alone does not establish a greater treatment benefit. Special caution remains warranted in patients aged ≥ 75 years, in whom renal function is frequently overestimated by serum-creatinine-based formulae; estimating creatinine clearance with the Cockcroft–Gault equation is advisable before initiating therapy, given that accumulation has been implicated in the rare extrapyramidal adverse events [12].

### 6.3. Microvascular Angina and Heart Failure Phenotypes

Persistent chest pain in the absence of obstructive epicardial disease (ANOCA/INOCA) is increasingly attributed to coronary microvascular dysfunction and accounts for a substantial fraction of refractory angina, particularly in women [1,54,55,56]. Women are disproportionately represented among patients with ANOCA/INOCA and coronary microvascular dysfunction, and actual ESC guidelines emphasize a structured, endotype-based diagnostic and therapeutic approach in this population. Although trimetazidine’s non-hemodynamic metabolic mechanism provides a plausible rationale when angina persists, no adequately powered trial has established a sex-specific benefit or defined its role specifically in women with INOCA. Accordingly, its use in this setting should be individualized and symptom-directed [57]. The PATMOS study by Ilic et al. examined trimetazidine’s effect on the coronary microcirculation after stenting and reported measurable improvements in microvascular indices, including coronary flow reserve [22]. Structured, stratified management of these endotypes—exemplified by the CorMicA trial—has been shown to improve angina status in patients without obstructive disease [57]. Although the trial was small and mechanistic in design, the findings support the biological plausibility of a role for metabolic modulation in microvascular angina, an area where therapeutic options remain limited. In heart failure, the Fragasso trial [17] and subsequent meta-analyses have suggested benefits on LVEF and functional capacity in both ischemic and non-ischemic phenotypes, although the evidence in heart failure with preserved ejection fraction (HFpEF) is still preliminary, comes from small heterogeneous studies [58], and should not be extrapolated to clinical practice until adequately powered trials are completed.

### 6.4. Post-Percutaneous Coronary Intervention

ATPCI is the largest and most contemporary randomized evaluation of trimetazidine in CAD [3,21]. Six thousand and seven patients at 365 centers in 27 countries were randomized to trimetazidine 35 mg twice daily or placebo on top of optimal medical therapy after successful PCI (elective in 58% and for acute coronary syndrome in 42%). After a median follow-up of 47.5 months, the rates of the composite primary endpoint—cardiac death, hospitalization for cardiac events, recurrence or persistence of angina requiring an addition, switch, or up-titration of antianginal therapy, or recurrence or persistence of angina requiring coronary angiography—were virtually identical between groups (700 [23.3%] vs. 714 [23.7%]; hazard ratio 0.98, 95% CI: 0.88–1.09; *p* = 0.73). Pre-specified subgroup analyses—by age, sex, diabetes, prior myocardial infarction, indication for PCI, and country—were consistent with the overall null result. The drug was safe; it simply did not change hard outcomes. The principal implication is that any role for trimetazidine after PCI should be symptom-directed rather than prognostic; ATPCI does not establish a role for routine post-PCI use to improve cardiovascular outcomes [21]. Earlier mechanistic work by Danchin [59], together with controlled studies of peri-procedural ischemia during coronary angioplasty [60], and a smaller meta-analysis by Saroca et al. in patients undergoing reperfusion strategies described short-term signals (reductions in peri-procedural myocardial injury and troponin release) that have not been replicated in modern, adequately powered trials [61]. Notably, an analogous outcome trial of ranolazine in incompletely revascularized patients after PCI (RIVER-PCI) was likewise neutral [62].

## 7. Safety and Tolerability Profile

The non-hemodynamic mechanism of trimetazidine translates directly into a clean cardiovascular safety profile. The drug does not lower blood pressure, slow the heart, or depress contractility—all useful properties in a patient population that already tolerates poorly any further titration of β-blockers or CCBs [28]. ATPCI, designed with safety as a co-primary concern, confirmed good tolerability over a median follow-up of 47.5 months: serious adverse events did not differ significantly between groups, and there was no signal for proarrhythmia, QT prolongation, or worsening renal function [21].

Long-term safety has not been entirely uneventful, however. Post-marketing reports have described rare extrapyramidal adverse effects: parkinsonism, restless-legs syndrome, gait abnormalities, and tremor [63,64]. The mechanism is thought to involve mild dopaminergic antagonism via the piperazine moiety of trimetazidine, with accumulation in renal impairment and in older adults as the principal risk factors. Events are uncommon and largely reversible after withdrawal—typical resolution occurs within four months in most published case series—but they were sufficient to prompt the 2012 EMA Article 31 referral [12]. The referral restricted the indication to second-line treatment of stable angina, withdrew the non-cardiac indications (vertigo, tinnitus, visual disorders), introduced contraindications in Parkinson’s disease, Parkinsonian symptoms, tremor, restless-legs syndrome, and other related movement disorders, and recommended dose reduction in moderate renal impairment (CrCl 30–60 mL/min) with outright avoidance below CrCl 30 mL/min. Hepatic adverse events are very rare and idiosyncratic; routine monitoring of liver enzymes is not currently mandated [7,12].

The largest longitudinal study that provides evidence for TMZ-induced Parkinsonism comes from Kim et al., which evaluated its incidence in 9712-treated patients compared with 29,116 control patients for a 14-year timeframe. The incidence of drug-induced Parkinsonism was significantly higher in TMZ users (9.34 vs. 6.71 per 1000 patient-years, *p* < 0.0001) and TMZ use increased the risk significantly (aHR = 1.38; 95% confidence interval = 1.26–1.51). Compared to the population who were aged under 50 years, those aged over 50 years were more likely to develop parkinsonism (aHR = 2.48; 95% CI = 2.18–2.81 for age 50–64 years, and aHR = 3.22; 95% CI = 2.81–3.68 for age 65 years and older). Diabetes, ESRD, and stroke, higher cumulative doses of TMZ, and concurrent medications such as typical and atypical antipsychotics, prokinetics, and CCBs were significant predictors of a new diagnosis of parkinsonism. However, no data regarding TMZ discontinuation were reported [65].

These findings are concordant with the data reported by Kwon et al., who also identified TMZ use as an independent predictor of new-onset parkinsonism (aOR 1.39; 95% CI 1.06–1.81; *p* = 0.016). Similarly, age (aOR 2.07; 95% CI 1.13–3.74; *p* = 0.017) and stroke (aOR 3.23; 95% CI 1.87–5.61; *p* < 0.001) were associated with an increased risk of TMZ-induced Parkinsonism [66]. A systematic review by Dy et al. identified TMZ-induced Parkinsonism in 88 patients, manifested as akinesia, rigidity, postural disturbances, and gait disorders, reversible with drug withdrawal. Masmoudi et al. reported in a 21-case series typical parkinsonism, gait disorders, and restless leg syndrome that had complete resolution in 76% of cases (16/21) or partial improvement (5/21) after TMZ withdrawal [67].

A separate consideration relevant to physically active patients is the inclusion of trimetazidine on the World Anti-Doping Agency Prohibited List from 2014 onward, in class S4.4 (hormone and metabolic modulators) [68]. The listing applies in- and out-of-competition and prohibits any therapeutic use in elite athletes without an approved therapeutic use exemption (TUE). This is particularly relevant in young patients with anomalous coronary anatomy or congenital heart disease who may also be competitive athletes, in whom the prescription of trimetazidine has clear regulatory implications. European drug-utilization studies have shown sharp declines in dispensing after the 2012 and 2014 alerts [69], indicating that prescribing patterns have shifted toward more selective use in the patients for whom the benefit–risk balance is clearest.

## 8. Current Evidence and Guideline Position

The evidence base for trimetazidine has matured from early mechanistic work to large network meta-analyses and a multinational, double-blind, placebo-controlled outcome trial. The Danchin synthesis [13] is the anchor: it established that the drug is roughly equivalent in symptomatic effect to other non–heart-rate-lowering antianginals. Subsequent meta-analyses have not substantially altered that conclusion [14,15,34]. Practical guidance on formulation choice has been provided by Nagy et al. [33], who reviewed the comparative pharmacokinetics of immediate-release, modified-release twice-daily, and the newer once-daily modified-release preparations.

The guideline opinion has moved less smoothly. The 2024 ESC guidelines on chronic coronary syndromes [1] place trimetazidine at class IIb (level B), recommending it as a drug that “may be considered” when first-line therapy is insufficient. ATPCI [21] is now the dominant data point in the appraisal: it confirmed safety but did not show a reduction in MACE on top of optimal medical therapy after PCI. The 2023 AHA/ACC guideline on chronic coronary disease [70] does not include trimetazidine, primarily because the drug has never been approved by the U.S. Food and Drug Administration, which limits the geographic generalizability of any single recommendation. The clinical reading that follows from the available evidence is simple: trimetazidine relieves symptoms; it does not prevent events.

## 9. Why Was Trimetazidine Downgraded in the ESC Guidelines?

The 2024 downgrade from class IIa to class IIb may be interpreted in more than one way. The evidence on hard outcomes is weak, and the safety signal of rare extrapyramidal effects justifies caution. Three observations support this reading: ATPCI was the largest and best-designed contemporary trial and was neutral on MACE [21]; the EMA referral [12] restricted use, withdrew non-cardiac indications and made the safety profile more explicit; and modern ESC documents increasingly reward agents with proven survival or event-reducing benefit (statins [38], renin–angiotensin system blockers, SGLT2 inhibitors [39,40], GLP-1 receptor agonists [41], PCSK9 inhibitors [71]) and downgrade agents whose effect is purely symptomatic.

A complementary interpretation is that the symptomatic evidence base has not materially expanded since 2019, whereas the contemporary evidence informing overall benefit–risk assessment has evolved. The symptomatic evidence base for trimetazidine has not changed materially since 2019. The Danchin meta-analysis [13] still stands, while ATPCI did not show that the drug fails to relieve angina—it showed that adding it on top of optimal post-PCI therapy does not reduce major acute cardiovascular events, which is a different and weaker claim. Other second-line agents fared differently in the same document: ranolazine retains class IIa [42,43], supported by a more favorable regulatory profile and a broader body of recent symptomatic data, and ivabradine retains class IIa in symptomatic patients with sinus rhythm and an elevated heart rate [72]. Several commentaries have pointed out that the trimetazidine downgrade was made “with no new positive or negative evidence on symptoms”, suggesting that what changed was the committee’s standard for granting strong recommendations rather than the underlying symptomatic data.

Both interpretations are partially correct. The conservative interpretation reflects an internally consistent prognosis-first philosophy applied to a drug that has never been a survival therapy. The skeptical interpretation reflects the legitimate observation that purely symptomatic agents are now being judged against a standard they were never designed to meet—a standard that, if applied uniformly, would also push long-acting nitrates and several other symptomatic options out of strong recommendation status, although this is an argument from internal consistency rather than direct evidence of efficacy. The absence of new symptom-specific evidence should not be interpreted as evidence of sustained benefit in contemporary practice. Overall, the class IIb recommendation may partly reflect a broader guideline emphasis on therapies with demonstrated prognostic benefit; however, this remains an interpretation rather than a directly testable conclusion. Alternative explanations, including the neutral ATPCI result in post-PCI patients, limited contemporary symptomatic evidence, and regulatory safety restrictions, are equally plausible and support a cautious, symptom-directed role for trimetazidine. The 2024 ESC class IIb recommendation may reflect, at least in part, an evolving guideline emphasis on prognostic benefit; the neutral ATPCI result and safety-related restrictions are additional plausible contributors [73].

## 10. Practical Use of Trimetazidine

A potential candidate for trimetazidine is a patient with CCS and persistent effort angina despite maximally tolerated first-line antianginal therapy, particularly when further hemodynamic up-titration is limited by hypotension, bradycardia, or treatment-related adverse effects. Diabetic patients with diffuse coronary disease, older patients with limited hemodynamic reserve, and patients with persistent symptoms after anatomically successful PCI have been studied in selected trials or subgroups; however, available evidence does not establish that these groups derive a reliably greater benefit than other symptomatic patients [19,20,24,52,74]. In patients with suspected microvascular angina, the non-hemodynamic metabolic mechanism provides a plausible rationale, but clinical evidence remains limited [22].

Trimetazidine is contraindicated in patients with Parkinson’s disease, restless legs syndrome, or other movement disorders, owing to the risk of exacerbating extrapyramidal symptoms [12]. TMZ should also be avoided in patients with severe renal impairment (creatinine clearance < 30 mL/min), in whom drug accumulation increases the likelihood of neurological adverse events. Prescribing should be particularly cautious in patients already exposed to polypharmacy, where the incremental symptomatic benefit is expected to be modest and the risk of compromising adherence is substantial. Finally, competitive athletes must be informed that trimetazidine is included on the World Anti-Doping Agency (WADA) Prohibited List as a metabolic modulator [68].

**Figure 3:**
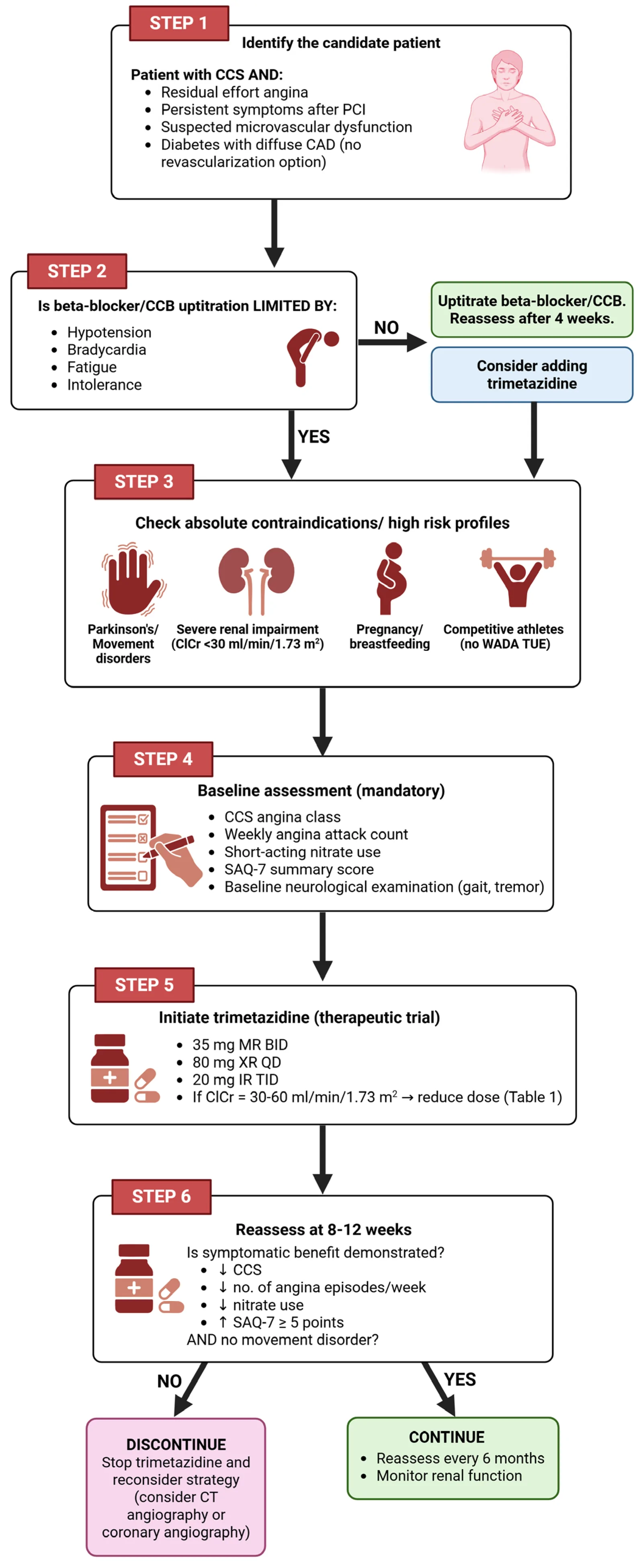
Proposed clinical decision algorithm for initiating, monitoring, and discontinuing trimetazidine in patients with chronic coronary syndrome. The algorithm operationalizes the indications, contraindications, and time-limited trial concept discussed in the text. CCS, Canadian Cardiovascular Society angina class; CrCl, creatinine clearance; SAQ-7, Seattle Angina Questionnaire-7; WADA, World Anti-Doping Agency. Created in BioRender. Tirziu, A. (2026) https://BioRender.com/fs28ukm (Accessed on 20 July 2026).

A reasonable evidence-informed approach is to initiate trimetazidine as a time-limited therapeutic trial of 8–12 weeks, with pre-specified assessment of symptoms (CCS class, weekly angina frequency, short-acting nitrate use and, where feasible, SAQ-7) and active surveillance for movement disorders. In the absence of a clear symptomatic benefit after approximately three months, discontinuation should be considered. From a system perspective, trimetazidine remains a low-cost generic in most European markets, which has historically supported broad use. Following the EMA restrictions, dispensing has declined substantially across Europe, and more selective prescribing may help align use with the current benefit–risk framework. A simple decision algorithm for clinical practice is summarized in Figure 3.

## 11. Limitations and Future Directions

Several limitations of the existing evidence and of the present synthesis should be stated plainly. This is a narrative review, not a systematic one. Accordingly, the PRISMA-style flow diagram is a transparency tool and should not be construed as evidence of a systematic review. The protocol was not prospectively registered (e.g., in PROSPERO), no formal risk-of-bias instrument was applied across all included studies, and quantitative pooling was not attempted. English-only inclusion excluded a sizeable Chinese-language literature on trimetazidine and is the most likely source of bias in our synthesis, although the direction of that bias is uncertain. The underlying trials are heterogeneous in size, era, dose, formulation, background therapy, and endpoints, and a substantial fraction predate high-intensity lipid-lowering therapy, contemporary PCI, SGLT2 inhibitors, GLP-1 receptor agonists, and, where clinically indicated, PCSK9 inhibitors. The data on ventricular remodeling come mostly from small single-center studies with surrogate echocardiographic endpoints. Safety information on extrapyramidal effects comes almost entirely from pharmacovigilance data and regulatory communications, with the under-reporting limitations that this entails.

Where should new evidence be generated? Six priorities seem most useful. First, a contemporary outcome trial of trimetazidine on top of full guideline-directed therapy—high-intensity statins, renin–angiotensin system blockade, SGLT2 inhibitors, and, where indicated, GLP-1 receptor agonists—is needed before any prognostic claim can be sustained. Second, mechanistic enrichment trials in phenotypes biologically suited to a metabolic intervention (microvascular angina, ischemic cardiomyopathy, diabetic coronary disease, HFpEF) would clarify whether targeted use is justified. Third, cardiac magnetic resonance imaging and positron emission tomography substudies, with quantitative perfusion and ^18^F-FDG metabolic imaging, could help establish biomarkers of metabolic responsiveness and individualize selection. Fourth, head-to-head randomized comparisons with ranolazine and long-acting nitrates would inform second-line sequencing, an evidence gap that current guidelines cannot fill. Fifth, prospective pharmacovigilance work in older adults and in patients with reduced renal function should quantify the true incidence of extrapyramidal symptoms and define safer dosing. Sixth, the role of trimetazidine in the peri-procedural window of PCI and in acute coronary syndromes deserves further large, well-designed trials, given the persistent signal from earlier mechanistic work [59,60,61,62].

Until such evidence arrives, trimetazidine may be considered as a second-line, symptom-directed adjunct for patients with residual angina in whom first-line therapy is insufficient or limited by tolerability, provided contraindications are excluded and response is reassessed.

## 12. Conclusions

Trimetazidine is a metabolic anti-ischemic agent with a clearly defined mechanism, a modest but reproducible symptomatic benefit, and a favorable hemodynamic profile, supports its consideration as add-on therapy when conventional drugs have reached their tolerability limits. Smaller studies suggest benefits on left ventricular function in ischemic cardiomyopathy and in selected high-risk subgroups, but the bulk of that evidence predates modern revascularization and contemporary secondary prevention. The ATPCI trial showed that the drug does not reduce major cardiovascular events when added to optimal post-PCI therapy, and the EMA restrictions, together with the rare but real extrapyramidal safety signal, define the boundaries of appropriate use. Importantly, because much of the symptomatic evidence predates contemporary secondary prevention and revascularization, the magnitude of benefit reported in historical trials may not be fully transferable to patients receiving optimized current care. The 2024 ESC class IIb recommendation may reflect an evolving guideline philosophy that increasingly favors prognostic over purely symptomatic agents, rather than new negative evidence on symptoms. Within those boundaries, trimetazidine may retain a narrower, symptom-directed role for patients with residual angina in whom further hemodynamic up-titration is limited or poorly tolerated.

## Figures and Tables

**Table 1:** Summary of pivotal trials and meta-analyses informing the use of trimetazidine in chronic coronary syndrome, with qualitative assessment of methodological quality.

Study (Year) [Ref]	Design	Population	Key Outcome	Findings/Quality
Danchin (2011) [13]	Network meta-analysis (218 trials, 19,028 pts)	Stable angina pectoris	Exercise duration; angina frequency	+46 s exercise duration vs placebo; comparable to other non–HR-lowering agents. High-quality synthesis; some included short and small trials.
Marzilli & Klein (2003) [14]	Meta-analysis of RCTs	Stable angina	Symptom and exercise outcomes	Significant improvement in exercise capacity and symptoms. Moderate quality.
Peng (2014) [15]	Meta-analysis of 13 RCTs (n = 1628)	Stable angina pectoris	Exercise tolerance; angina frequency	↓ 0.95 attacks/week; ↓ 0.98 nitrates/week; +49.8 s. Moderate quality; possible publication bias.
Brottier (1990) [16]	RCT (n = 20), 6-mo, double-blind	Severe ischemic cardiomyopathy	Symptoms; complementary therapy	Improved dyspnea (*p* < 0.001); ↓ residual angina. Small sample; hypothesis-generating.
Fragasso (2006) [17]	RCT (n = 55), 13-mo	Heart failure (mainly ischemic)	LVEF; LV volumes	LVEF 36→43% (*p* = 0.002); ↓ LVESV (*p* = 0.04). Single center; surrogate endpoints.
Belardinelli (2001) [18]	Prospective controlled study	Ischemic cardiomyopathy	Contractile reserve on dobutamine	Improved regional wall motion and contractile reserve. Small; mechanistic.
Xu (2014) [19]	RCT, double-blind, 2-yr	510 elderly multivessel CAD + DM after DES	Recurrent angina; LV structure	↓ angina (*p* = 0.024); preserved LV (all *p* < 0.01). Single-center; external validity limited.
TIGER, Sellier (2003) [20]	Prospective trial	Geriatric stable angina	Efficacy and tolerability	Favorable response and tolerability in elderly. Moderate quality.
ATPCI, Ferrari (2020) [21]	RCT, double-blind, n = 6007	Post-PCI on optimal therapy	MACE (composite primary)	HR 0.98 (95% CI 0.88–1.09; *p* = 0.73); well tolerated. High-quality, contemporary, neutral on outcomes.
PATMOS, Ilic (2023) [22]	Mechanistic RCT	Post-stenting CAD	Coronary microcirculation	Improvement in microvascular indices. Small; mechanistic.
COMBINE Angina, Marzilli (2026) [23]	Prospective observational (n = 578)	Recently diagnosed stable angina	SAQ-7; CCS class	SAQ-7 39→78 over 4 mo. (*p* < 0.0001); 0.7% AE. Observational; subject to selection and reporting bias.
Lin (2020) [24]	Meta-analysis of RCTs	Diabetic CAD	Cardiovascular outcomes	Beneficial effect in diabetic CAD population. Moderate; Chinese-language trials predominate.

AE, adverse event; CAD, coronary artery disease; CCS, Canadian Cardiovascular Society (column 4) and chronic coronary syndrome (in text); DES, drug-eluting stent; DM, diabetes mellitus; HR, hazard ratio; LV, left ventricular; LVEF, left ventricular ejection fraction; LVESV, left ventricular end-systolic volume; MACE, major adverse cardiovascular events; pts, patients; RCT, randomized controlled trial; SAQ-7, Seattle Angina Questionnaire-7.

**Table 2:** Qualitative certainty assessment of the current studies on TMZ use in CAD.

Clinical Domain	Qualitative Certainty	Rationale
Symptom relief and exercise tolerance [13,14,15,16]	Moderate	Consistent direction of effect across older RCTs and meta-analyses; heterogeneity and limited applicability to contemporary care reduce certainty.
Health status/quality of life [16,23]	Low to moderate	Supportive patient-reported outcomes, but limited contemporary blinded randomized evidence; COMBINE Angina is observational.
LV function/remodeling [17,18,19]	Low	Small, heterogeneous, largely single-center trials using surrogate echocardiographic endpoints.
Major cardiovascular outcomes after PCI [21]	High certainty of no demonstrated benefit	ATPCI was large, contemporary, randomized, and neutral; its population does not establish effects in every CCS phenotype.
Diabetes, older age, and post-PCI subgroups [19,20,24]	Low	Predominantly single-center, subgroup, or observational data with limited external validity.
INOCA/microvascular dysfunction, including women [22]	Very low	Limited mechanistic and small-study evidence; no established sex-specific treatment effect.

Note: Certainty categories represent an author-derived narrative appraisal based on study design, consistency, directness, precision, risk of bias, and applicability. They are not formal GRADE ratings.

**Table 3:** Recommended trimetazidine dosing by renal function and key contraindications.

Renal Function (CrCl)	Immediate-Release (IR)	Modified-Release (MR)
Normal (≥60 mL/min)	20 mg three times daily	35 mg twice daily (or 80 mg once daily)
Moderate impairment (30–60 mL/min)	20 mg twice daily	35 mg once daily
Severe impairment (<30 mL/min)	Contraindicated	Contraindicated
Parkinson disease/movement disorders	Contraindicated	Contraindicated

*CrCl*, *creatinine clearance*.

## Data Availability

No new data were created or analyzed in this study. Data sharing is not applicable to this article.

## Supplementary Materials

The following supporting information can be downloaded at https://www.mdpi.com/article/10.3390/ph19091370/s1. The literature search protocol, inclusion and exclusion criteria and screening counts can be accessed in the Supplementary Material S1.

## Abbreviations

The following abbreviations are used in this manuscript:

Abbreviation	Full term
ACEi	Angiotensin-converting enzyme inhibitor
ACS	Acute coronary syndrome
AHA/ACC	American Heart Association/American College of Cardiology
ANOCA/INOCA	Angina/ischemia with non-obstructive coronary arteries
ARB	Angiotensin receptor blocker
ATPCI	Efficacy and safety of Trimetazidine in patients with angina pectoris having been treated by Percutaneous Coronary Intervention
CAD	Coronary artery disease
CCB	Calcium channel blocker
CCS	Chronic coronary syndrome/Canadian Cardiovascular Society
CI	Confidence interval
CMR	Cardiac magnetic resonance imaging
CrCl	Creatinine clearance
CrI	Credibility interval
EMA	European Medicines Agency
ESC	European Society of Cardiology
GLP-1	Glucagon-like peptide-1
HFpEF	Heart failure with preserved ejection fraction
HFrEF	Heart failure with reduced ejection fraction
HR	Hazard ratio
LVEF	Left ventricular ejection fraction
MACE	Major adverse cardiovascular events
MCID	Minimal clinically important difference
PCI	Percutaneous coronary intervention
PCSK9	Proprotein convertase subtilisin/kexin type 9
pFOX	Partial fatty acid oxidation
RCT	Randomized controlled trial
SAQ-7	Seattle Angina Questionnaire-7
SGLT2	Sodium–glucose cotransporter 2
TMZ	Trimetazidine
TUE	Therapeutic use exemption
WADA	World Anti-Doping Agency
3-KAT	Long-chain 3-ketoacyl coenzyme A thiolase
