# Supplementary material for: Trimetazidine in Chronic Coronary Syndrome and Coronary Artery Disease: A Narrative Review of Clinical Efficacy, Safety, and Current Guideline Position
Source: Pharmaceuticals (Basel). 2026 Aug 29;19(9):1370. doi: 10.3390/ph19091370 (PMC13610195; doi:10.3390/ph19091370)
Supplement: Supplementary file 1 [file pharmaceuticals-19-01370-s001.zip › pharmaceuticals-4493113-supplementary.pdf]

Supplementary Material S1. Database search strategy and screening counts

Manuscript: Trimetazidine in Chronic Coronary Syndrome and Coronary Artery Disease: A Narrative Review of Clinical Efficacy, Safety, and Current Guideline Position

Authors: Daniel Miron Brie, Cristian Mornoș, Alexandru Tîrziu, Roxana Popescu and Alina Diduța Brie

S1.1. Overview of the literature search

A structured search of PubMed/MEDLINE, Embase, and the Cochrane Central Register of Controlled Trials (CENTRAL) was conducted between January 1990 and March 2026. Reference lists of all included articles and prior reviews were screened to identify additional eligible studies, and international clinical practice guidelines (ESC, AHA/ACC, NICE) and regulatory communications (EMA, ANSM, MHRA) were also examined. The search was last updated on 11 March 2026. No language filter was applied at the search step, but non-English publications were excluded at the screening step; this decision is acknowledged in the Limitations section of the main manuscript.

Titles and abstracts were screened independently by two authors (D.M.B. and A.D.B.). Potentially eligible full-text articles were assessed by both reviewers. Disagreements that could not be resolved by discussion were adjudicated by a third reviewer (C.M.). The protocol was not prospectively registered.

S1.2. PubMed/MEDLINE search string (full)

The complete PubMed search string used to identify candidate articles was as follows. The string is reproduced verbatim and can be pasted into the PubMed Advanced Search Builder to reproduce the search:

```
(
    "trimetazidine"[MeSH Terms]
    OR "trimetazidine"[Title/Abstract]
    OR "vastarel"[Title/Abstract]
    OR "preductal"[Title/Abstract]
)
AND
(
    "angina pectoris"[MeSH Terms]
    OR "angina, stable"[MeSH Terms]
    OR "coronary artery disease"[MeSH Terms]
    OR "myocardial ischemia"[MeSH Terms]
    OR "heart failure"[MeSH Terms]
    OR "percutaneous coronary intervention"[MeSH Terms]
```

```
    OR "chronic coronary syndrome"[Title/Abstract]
    OR "chronic coronary syndromes"[Title/Abstract]
    OR "stable angina"[Title/Abstract]
    OR "stable coronary"[Title/Abstract]
    OR "ischemic cardiomyopathy"[Title/Abstract]
    OR "ischaemic cardiomyopathy"[Title/Abstract]
    OR "PCI"[Title/Abstract]
    OR "percutaneous coronary intervention"[Title/Abstract]
    OR "microvascular angina"[Title/Abstract]
    OR "ANOCA"[Title/Abstract]
    OR "INOCA"[Title/Abstract]
)
AND
(
    "humans"[MeSH Terms]
)
AND
(
    ("1990/01/01"[Date - Publication] : "2026/03/31"[Date - Publication])
)
```

Equivalent searches were constructed for Embase (using EMTREE terms 'trimetazidine'/exp AND ('angina pectoris'/exp OR 'coronary artery disease'/exp OR 'heart failure'/exp OR 'percutaneous coronary intervention'/exp)) and for the Cochrane Central Register of Controlled Trials. Filters for study design were not applied at the search step to maximize sensitivity; instead, design-based filtering was applied during screening.

S1.3. Inclusion and exclusion criteria

S1.3.1. Inclusion criteria

Population: Adults (≥ 18 years) with stable angina, chronic coronary syndrome, coronary artery disease, ischemic cardiomyopathy, or ischemic heart failure. Studies enrolling at least 50 patients were eligible.

Intervention: Trimetazidine in any formulation (immediate-release, modified-release twice-daily, modified-release once-daily) at standard clinical doses, used alone or as add-on therapy.

Comparator: Placebo, no treatment, or active comparator (other antianginal agents). Single-arm studies without a comparator were not eligible.

Outcomes: At least one of the following: exercise tolerance (total exercise duration, time to 1-mm ST depression, time to onset of angina); angina frequency; short-acting nitrate consumption; patient-reported outcomes (Seattle Angina Questionnaire or comparable instrument); left ventricular function or remodeling; major adverse cardiovascular events (MACE); all-cause or cardiovascular mortality.

Study design: Randomized controlled trials, conventional or network meta-analyses, systematic reviews, and large prospective observational cohorts.

Follow-up: Minimum follow-up of 3 months.

Language: English.

S1.3.2. Exclusion criteria

- Case reports and case series with fewer than 20 patients.
- Single-arm uncontrolled studies.
- Non-cardiac indications (vertigo, tinnitus, visual disorders, dizziness, Ménière disease).
- Trials without a clearly defined clinical or functional endpoint.
- Conference abstracts published only as posters and not as full-text articles, unless they represented the only available report of a pivotal trial (e.g., the Saroca meta-analysis).
- Animal and in vitro studies.
- Duplicate publications and overlapping cohorts (only the most complete or most recent report was retained).

S1.4. Screening counts (PRISMA-style)

The screening counts shown below populate Figure 1 in the main manuscript. Counts are reported by source and by reason for exclusion.

**Identification**	
PubMed/MEDLINE	612
Embase	487
Cochrane CENTRAL	74
Reference-list / hand search	21
Guidelines and regulatory communications	8
Total records identified	1,202
**Screening**	
Records after duplicates removed	1,058 (duplicates: 144)
Records excluded at title/abstract	921

• Off-topic / wrong indication	612
• Non-cardiac use	184
• Animal or in vitro only	125
Full-text articles assessed	137
**Eligibility**	
Full-text articles excluded	89
• Non-English publication	41
• Fewer than 50 patients	13
• Follow-up < 3 months	9
• Case report or small case series	8
• No eligible endpoint reported	7
• Single-arm / no comparator	6
• Duplicate or overlapping cohort	5
Studies included in qualitative synthesis	48
**Included**	
• RCTs (including ATPCI and COMBINE Angina)	24
• Meta-analyses and systematic reviews	9
• Observational / cohort studies	7
• Guidelines and regulatory documents	5
• Mechanistic / pharmacology reviews	3
References cited as pivotal evidence in the manuscript	25

S1.5. Note

Counts in Figure 1 and in the table above reflect the search and screening process at the cut-off date of 11 March 2026. Searches were last verified on 18 March 2026. No additional eligible records were identified between these dates. The full list of 48 studies retained in the qualitative synthesis is available from the corresponding author upon reasonable request; the 25 references actually cited in the main manuscript appear in the Reference List of the article.

Correspondence: alexandru.tirziu@umft.ro
